# Supplementary material for: From sequence to enzyme mechanism using multi-label machine learning
Source: BMC Bioinformatics. 2014 May 19;15:150. doi: 10.1186/1471-2105-15-150 (PMC4229970; doi:10.1186/1471-2105-15-150)
Supplement: Additional file 2 — Java code of ml2db. Additional file ml2db_code.tar.gz contains the Java source code to run the multi-label machine learning experiments and save the results to database. The code’s Javadoc is included. [file 1471-2105-15-150-S2.zip › additional file 2/ml2db/ml2db/doc/mulan/data/ConverterCLUS.html]

ConverterCLUS


JavaScript is disabled on your browser.


- Overview
- Package
- Class
- Use
- Tree
- Deprecated
- Index
- Help

- Prev Class
- Next Class

- Frames
- No Frames

- All Classes

- Summary:
- Nested |
- Field |
- Constr |
- Method

- Detail:
- Field |
- Constr |
- Method


mulan.data

## Class ConverterCLUS

- java.lang.Object
- - mulan.data.ConverterCLUS

- ---

    

  ```
  public class ConverterCLUS
  extends java.lang.Object
  ```

  Class that converts a dataset that is originally in the format of the
  Clus system to a format
  that is suitable for Mulan. An arff and an xml file are created.

  The arff file contains the original dataset with all labels converted to
  separate attributes and properly converted instances. The xml file contains
  the hierarchy of the labels.

  Version:
  :   2012.02.06

  Author:
  :   George Saridis, Grigorios Tsoumakas

- - ### Constructor Summary

    Constructors

    Constructor and Description

    `ConverterCLUS()`
  - ### Method Summary

    Methods

    | Modifier and Type | Method and Description |
    | --- | --- |
    | `static void` | `convert(java.lang.String sourceFilename, java.lang.String arffFilename, java.lang.String xmlFilename)` Converts the original dataset to mulan compatible dataset. |

    - ### Methods inherited from class java.lang.Object

      `equals, getClass, hashCode, notify, notifyAll, toString, wait, wait, wait`

- - ### Constructor Detail


    - #### ConverterCLUS

      ```
      public ConverterCLUS()
      ```
  - ### Method Detail


    - #### convert

      ```
      public static void convert(java.lang.String sourceFilename,
                 java.lang.String arffFilename,
                 java.lang.String xmlFilename)
                          throws java.lang.Exception
      ```

      Converts the original dataset to mulan compatible dataset.

      Parameters:
      :   `sourceFilename` - the source file name
      :   `arffFilename` - the converted arff name
      :   `xmlFilename` - the xml name

      Throws:
      :   `java.lang.Exception`


- Overview
- Package
- Class
- Use
- Tree
- Deprecated
- Index
- Help

- Prev Class
- Next Class

- Frames
- No Frames

- All Classes

- Summary:
- Nested |
- Field |
- Constr |
- Method

- Detail:
- Field |
- Constr |
- Method
